# Positive end-expiratory pressure setting based on transpulmonary pressure during robot-assisted laparoscopic prostatectomy: an observational intervention study

**DOI:** 10.1186/s40981-022-00501-y

**Published:** 2022-02-12

**Authors:** Koichi Nakazawa, Ami Kodaira, Rika Matsumoto, Tomoko Matsushita, Ryotaro Yoshikawa, Yusuke Ishida, Hiroyuki Uchino

**Affiliations:** 1 Department of Anesthesia, Tokyo Medical University Hospital, 6-7-1 Nishishinjyuku, Shinjyuku-ku, Tokyo, 1600023 Japan; 2 Department of Anesthesia, Mitsui Memorial Hospital, Kanda-Izumi-cho 1, Chiyoda-ku, Tokyo, 101-8643 Japan

**Keywords:** Robot-assisted laparoscopic prostatectomy, Driving pressure, Respiratory system compliance, PEEP, Transpulmonary pressure

## Abstract

**Background:**

In robot-assisted laparoscopic prostatectomy (RALP), concerns include the formation of atelectasis and reduced functional residual capacity. The present study aimed to examine the feasibility of positive end-expiratory pressure (PEEP) setting based on transpulmonary pressure (Ptp) as well as the effects of incremental PEEP on respiratory mechanics, blood gases, cerebral oxygenation (rSO_2_), and hemodynamics.

**Methods:**

Fourteen male patients who were scheduled to receive RALP were recruited. Patients received mechanical ventilation (tidal volume of 6 mL kg^−1^) and were placed in Trendelenburg position with positive-pressure capnoperitoneum. PEEP levels were increased from 0 to 15 cmH_2_O (5 cmH_2_O per increase) every 30 min. PEEP levels were assessed where end-expiratory Ptp levels of ≥0 cmH_2_O were achieved (PtpEEP0). Airway pressure, esophageal pressure, cardiac index, and blood gas and rSO_2_ values were measured after 30 min at each PEEP step and respiratory mechanics were calculated.

**Results:**

With increasing PEEP levels from 0 to 15 cmH_2_O or PtpEEP0, the values of PaO_2_ and respiratory system compliance increased, and the values of driving pressure decreased. The median PEEP level associated with PtpEEP0 was 15 cmH_2_O. Respiratory system compliance values were higher at PtpEEP0 than those at PEEP5 (*P* = 0.02). Driving pressure was significantly lower at PtpEEP0 than at PEEP5 (*P* = 0.0036). The cardiac index remained unchanged, and the values of rSO_2_ were higher at PtpEEP0 than at PEEP0 (right; *P* = 0.0019, left; *P* = 0.036).

**Conclusions:**

PEEP setting determined by transpulmonary pressure can help achieve higher respiratory system compliance values and lower driving pressure without disturbing hemodynamic parameters.

## Background

Robotic surgery may enhance laparoscopic procedures in various contexts. However, respiratory management during robot-assisted laparoscopic prostatectomy (RALP) requires caution, as the procedure requires positive-pressure capnoperitoneum and steep Trendelenburg position. The dependent lungs may be compressed by positive pleural pressure, and their alveoli may collapse at end-expiration if positive end-expiratory pressure (PEEP) levels are inadequate. PEEP helps to prevent the formation of atelectasis and reduction of functional residual capacity; however, high airway pressure may result in overdistension or nonuniform ventilation distribution, or hemodynamic depression.

To reduce the risk of ventilator-induced lung injury and avoid barotrauma, evaluating the stress on the alveolar wall is recommended, while keeping the alveolus open [1]. Transpulmonary pressure (Ptp: airway pressure–intrathoracic pressure) has been previously used to determine PEEP levels in the respiratory management of acute respiratory distress syndrome [2, 3]. If the Ptp values at end-expiration are of <0 cmH_2_O, then the alveoli may collapse. Intrathoracic pressure can be substituted with esophageal pressure (Peso), measured with an esophageal balloon catheter. Intrathoracic pressure may be elevated during RALP; however, few studies to date have observed the changes in Ptp values at end-expiration with increasing PEEP levels. While low tidal volumes may benefit patients receiving general anesthesia using positive-pressure ventilation [4–8], the method for determining the optimum PEEP level during RALP remains unclear. Low PEEP may induce negative Ptp on end-expiration and promote atelectasis.

The present study aimed to clarify the changes in intrapleural and transpulmonary end-expiratory pressure (PtpEEP) values associated with increasing PEEP, given the positive-pressure capnoperitoneum combined with the Trendelenburg position, to determine the PEEP level where the Ptp exceeds 0 cmH_2_O. This study also aimed to examine the effects of those PEEP levels on cardiac output and cerebral oxygenation.

## Methods

This single-center, prospective, observational interventional study was approved by the Ethics Committee of Tokyo Medical University Hospital (T2018-0065). The study protocol adhered to the principles of the Declaration of Helsinki; written informed consent was obtained from all patients. This registered clinical trial (UMIN000036376) included consecutive patients undergoing the RARP procedure at the Tokyo Medical University Hospital from April to August in 2019.

We enrolled male patients with the American Society of Anesthesiologists (ASA) Physical Status Classification score of 1 or 2 points, scheduled for RARP using a robotic operating system (da Vinci™ Surgical System, Intuitive Surgical, Inc., Sunnyvale, CA, USA). Patients with a history of chronic obstructive pulmonary disease, renal or heart failure, esophageal diseases, or body mass index (BMI) of ≥35 kg m^−2^ were excluded from this study.

No premedication was administered. Upon arriving in the operating theater, patients were monitored by electrocardiography; non-invasive automated blood pressure measurement and pulse oximetry were performed. SedLine® Brain Function Monitoring for patient state index (PSi) and O3® Regional Oximetry sensors for bilateral forebrain oxygenation (rSO_2_) monitoring were attached to the forehead and connected to Root Monitor® (Masimo, Irvine, CA, USA). A pulse oximetry probe was placed on the forefinger; body temperature was measured, using a 3M™ BearHugger™ deep temperature monitoring system. The patients were breathing a fraction of inspired oxygen (F_I_O_2_) of 1.0 for 3 min before the induction of general anesthesia with intravenous remifentanil at a rate of 0.3 μg kg^−1^ min^−1^ and propofol via target-controlled infusion to a plasma concentration of 4–4.5 μg mL^−1^. The lungs were ventilated manually, using a Jackson Rees breathing system via a face mask with an F_I_O_2_ of 1.0. Muscle relaxation was achieved with rocuronium of 1 mg kg^−1^ to facilitate orotracheal intubation. The patients were intubated with a cuffed reinforced tracheal tube with an internal diameter of 7.5 mm, using a McGrath MAC® video laryngoscope (Medtronic Co., USA). After confirming tracheal intubation, the lungs were ventilated using a mechanical ventilator (AVEA™ Ventilator Systems; CareFusion Inc., San Diego, CA, USA) with volume-controlled ventilation. The inspiratory-to-expiratory time ratio was 1:2.5 with an end-inspiratory pause of 20% and tidal volume (VT) of 6 mL kg^−1^ of the predicted body weight. The predicted body weight was calculated as 49.9 + 0.91 × (height [cm] − 152.4).

The respiratory rate was initially set at 12 breaths/min and changed to maintain normocapnia (end-tidal carbon dioxide partial pressure [EtCO_2_] of 35–45 mmHg). Patients were ventilated using oxygen and air, with an F_I_O_2_ of 0.5. After the recruitment maneuver (35 cmH_2_O CPAP for 30 s), 3 cmH_2_O PEEP was added. A 22-G arterial cannula was inserted percutaneously into the radial artery after the induction of anesthesia for continuous monitoring of arterial pressure and blood gas sampling; it was also connected to a Flo Trach™ sensor with a Vigileo monitor system (Edwards Lifesciences, Irvine, CA, USA) for continuous monitoring of arterial pressure wave form, based on cardiac index (CI) and stroke volume variation (SVV). Arterial blood gas analysis was performed within 1 min of sampling, using a blood gas analyzer (The epoc® Blood Analysis System, Co. Siemens Healthineers, PA, USA). To measure esophageal pressure and gastric suctioning, a 16-Fr SmartCath™ adult nasogastric tube with an esophageal balloon (Vyaire Medical Inc., Mettawa, IL, USA) was inserted transorally and advanced into the stomach. By pulling back the tube, the positioning of the balloon was confirmed in the presence of a cardiac oscillation reflective of cardiac activity and esophageal pressure wave fluctuation during a ventilation cycle. Furthermore, the balloon location was confirmed using an occlusion technique; the airway was occluded at end-expiration and esophageal pressure and airway pressure were simultaneously compared for similarity. The position of the catheter was also checked on routine postoperative chest radiography.

After the induction of anesthesia, patients were placed in a horizontal lithotomy position. Anesthesia was maintained with a continuous infusion of propofol, remifentanil, and rocuronium throughout the surgery. The infusion rate of propofol was adjusted to maintain the PSi in the range of 25–50, with the administration of remifentanil at the rate of 0.3 μg kg^−1^ min^−1^. The neuromuscular block was maintained by continuous intravenous administration of rocuronium at the rate of 0.3 mg kg^−1^ h^−1^. Hemodynamic stability (arterial systolic pressure and heart rate of 80–100% of the preanesthetic value) was maintained by fluid management or vasopressors. Specifically, standardized fluid management was performed using acetated Ringer’s solution. In cases of intraoperative hypotension (mean arterial pressure of <65 mmHg or a decrease in mean arterial pressure by >20% from baseline), 6% hydroxyethyl starch in 0.9% sodium chloride solution was given, provided the SVV was >13%; otherwise, it was managed with phenylephrine or ephedrine, as required.

### Study protocol

The patient was placed in the 30° Trendelenburg lithotomy position with a positive-pressure capnoperitoneum and CO_2_ insufflation, when the respiratory rate increased to 15 breaths/min. Capnoperitoneum was kept at the pressure of 12 mmHg; however, pressure levels were occasionally changed at the surgeon’s discretion. PEEP level was initially maintained at 0 cmH_2_O for 30 min; thereafter, while the intraabdominal pressure was kept at 12 mmHg, blood gas levels and hemodynamic and ventilatory parameters were recorded. Esophageal and airway pressure values were simultaneously read on a monitor screen at end-inspiration and end-expiration. Ptp estimates for both phases were calculated, as the difference between airway and esophageal pressure values. The respiratory rate was changed, such that the ETCO_2_ levels were between 40 and 50 mmHg during surgery.

PEEP levels were increased by 5 cmH_2_O in a stepwise manner at 30-min intervals; measurements were performed before each increase. Recruitment maneuver was performed before each PEEP trial. Once the PEEP levels reached 15 cmH_2_O and the measurements were completed, PEEP levels were changed, such that the Ptp was 0 cmH_2_O (PtpEEP0).

### Data collection

Patient characteristics of interest included height, weight, body mass index, ASA physical status, and respiratory function test findings. The values of the following parameters of respiratory mechanics were recorded: inspiratory peak pressure, inspiratory plateau pressure (Pplat), end-inspiratory esophageal pressure (PesoEIP), end-expiratory esophageal pressure (PesoEEP), and ETCO_2_.

Hemodynamic parameters of interest included heart rate, mean arterial pressure, arterial pressure wave form base CI, and SVV values. The following arterial blood gases were examined: pH, PO_2_, PCO_2_, and SaO_2_. Cerebral oxygenation was measured as bilateral rSO_2_ levels.

The following parameters were calculated as follows: respiratory system driving pressure (DP) = Pplat − PEEP; end-inspiratory transpulmonary pressure (PtpEIP) = Pplat − PesoEIP; end-expiratory transpulmonary pressure (PtpEEP) = PEEP − PesoEEP; transpulmonary driving pressure (PtpDP) = PtpEIP − PtpEEP; respiratory system compliance = VT/(Pplat − PEEP); chest wall compliance = VT/(PesoEIP − PesoEEP); and pulmonary compliance = VT/ PtpDP.

Data were collected (1) after the induction of anesthesia (baseline), (2) 30 min after capnoperitoneum and Trendelenburg position (PEEP0 cmH_2_O: PEEP0) were achieved, (3) 30 min after PEEP of 5 cmH_2_O (PEEP5) was achieved, (4) 30 min after PEEP of 10 cmH_2_O was achieved (PEEP10), (5) 30 min after PEEP15 cmH_2_O (PEEP15) was achieved, (6) 30 min after PEEP level corresponding to PtpEEP of 0 (PtpEEP0) was achieved, and (7) at the end of surgery. After the RALP procedure was completed, the patients were returned to the supine position and PEEP levels were set at 5 cmH_2_O.

### Statistical analysis

The present study aimed to determine the PEEP levels, where the PtpEEP values exceeded 0 cmH_2_O during the RALP surgery, and to observe whether such PEEP levels were beneficial for oxygenation or respiratory mechanics parameters. A formal power analysis was not performed because this was an observational study; however, sample size calculation was performed based on the preliminary findings obtained from the first seven patients in the first 2 months. Since PtpDP from PEEP 0 to PtpEEP0 values were 9.3±5.8 cmH_2_O and 6.0±2.8 cmH_2_O, we calculated that 11 patients were required to test the null hypothesis at a significance level of 0.05 and power of 0.90. Accounting for possible study dropouts, we aimed to include 16 participants. Categorical variables were reported as counts and percentages. Continuous data were examined for normal distribution, using the Shapiro–Wilk test, and were presented as mean±standard deviation or median and interquartile range, as appropriate. The Friedman non-parametric test with Scheffe’s multiple comparison procedure was used to test for differences between each PEEP level. All analyses were performed using the statistical software package BellCurve for Excel for Windows® (Social Survey Research Information C., Ltd. Tokyo, Japan). *P*-values of <0.05 were considered statistically significant.

## Results

Written informed consent was obtained from 16 patients enrolled in the study. Two patients were excluded due to esophageal balloon catheter failure; a total of 14 patients were included (Table 1). No patient showed a deterioration in circulatory dynamics or received vasopressors during robot-assisted procedures. All robot-assisted procedures were successfully performed, and all patients completed this protocol without any episodes of clinical problems. No patient developed any perioperative complications related to positive-pressure capnoperitoneum, positioning, or anesthesia during and after surgery.

**Table 1:** Patient characteristics

Age (years)	63±6
Height (cm)	170±7
Weight (kg)	70.2±12.8
BMI (kg/m^2^)	24.1±3.8
Duration of anesthesia (min)	304±51
FEV1%	75.2±7.2
%VC	122.5±12.6

Data are presented as the mean±SD

*BMI* body mass index, *FEV1%* forced expiratory volume in the first second, *%VC* percent vital capacity

### Effects of PEEP on respiratory pressures and ventilatory mechanics

Ventilation pressure and respiratory mechanics parameter values at each PEEP level are presented in Table 2. The value of PesoEEP during the Trendelenburg position with positive-pressure capnoperitoneum at PEEP0 was 10 (6.8–14) cmH_2_O; it increased with the increases in PEEP levels. However, increasing PEEP from 0 to 15 cmH_2_O reduced the gap between PesoEEP and PEEP levels. PtpEEP of 0 was associated with the PEEP level of 15 (range 12–20) cmH_2_O.

**Table 2:** Changes in ventilation pressures and respiratory mechanics during anesthesia

	After induction	Positive-pressure capnoperitoneum and Trendelenburg position					End of surgery
		PEEP0	PEEP5	PEEP10	PEEP15	PtpEEP0	
TV (mL)	390 [382, 400]	390 [382, 400]	390 [382, 400]	390 [382, 400]	400 [382, 420]	395 [372, 420]	395 [372, 420]
RR (/min)	12	15 [15, 17]	16 [15, 18]	16 [15, 20]	18 [15, 20]^⁑0,*5^	18 [15, 20]^⁑0,*5^	15 [15, 16]
MV(L/min)	4.8 [4.8, 5.4]	6.72 [5.4, 7.2]	6.66 [5.7, 7.2]	6.75 [5.85, 7.29]	7.2 [6.3, 7.92]^*0^	7.2 [6, 8]	5.76 [5.55, 6.45]
PEEP (cmH_2_O)	3	0	5	10	15	15 [12, 16]	5
Pplat (cmH_2_O)	9 [9, 10]	14 [13, 18]	19 [16, 20]	23 [21, 24]^*0^	27 [25, 28]^⁑0,⁑5^	27 [25, 28]^⁑0,⁑5^	11 [10, 13]
DP (cmH_2_O)	6 [6, 7]	14 [13, 18]	14 [11, 15]	13 [11, 14]^⁑0^	12 [10, 13]^⁑0,*5^	12 [10, 13]^⁑0,⁑5^	6 [5, 8]
Respiratory system compliance (mL/cmH_2_O)	61 [51, 64]	24 [21, 25]	27 [22, 32]	32 [28, 35]^⁑0^	32 [29, 34]^⁑0^	33 [30, 35]^⁑0,*5^	60 [52, 65]
PesoEEP (cmH_2_O)	6 [5, 8]	10 [7, 14]	10 [9, 12]	12 [11, 15]	15 [13, 15]^⁑0,*5^	13 [12, 16]	6 [4, 8]
PesoEIP (cmH_2_O)	8.5 [7.3, 9.3]	17 [14.1, 20.1]	17.4 [14, 19.8]	19.4 [15.5, 21]	20.6 [17.8, 22.2]	19 [18.1, 21.2]^*5^	9.4 [7.2, 9.8]
PtpEEP (cmH_2_O)	−2.9 [−4.8, −1.4]	−9.8 [−11.7, −6.3]	−5.7 [−7.9, −4]	−3.1 [−4, −1.9]	0 [−0.75, 1]^⁑0,⁑5^	0 [0, 0.8]^⁑0,⁑5^	−1.2 [−2.8, 0]
PtpDP (cmH_2_O)	4.3 [3.2, 6.7]	9.3 [5.8, 10.7]	7.7 [4.7, 10.2]	5.9 [4, 9.2]^*0^	5.7 [3.9, 7.8]	5.3 [4, 6.7]	3.8 [2.5, 7]
Lung compliance (mL/cmH_2_O)	94 [57, 146]	43 [39, 63]	54 [43, 83]	64 [43, 95]^*0^	74 [58, 100]^*0^	83 [64, 100]^*0^	74 [50, 133]
Chest wall compliance (mL/cmH_2_O)	203 [147, 246]	65 [43, 74]	59 [51, 109]	64 [53, 87]	67 [60, 120]	60 [57, 74]	177 [130, 350]

Data are presented as median values [interquartile range]

*TV* tidal volume, *RR* respiratory rate, *MV* minute volume, *PEEP* positive expiratory pressure, *Pplat* inspiratory plateau pressure, *DP* driving pressure, *PesoEEP* end-expiratory esophageal pressure, *PesoEIP* end-inspiratory esophageal pressure, *PtpEEP* transpulmonary end-expiratory pressure, *PtpDP* transpulmonary driving pressure.

^*0^*P* < 0.05 vs PEEP0; ^*5^*P* < 0.05 vs PEEP5; ^⁑0^*P* < 0.01 vs PEEP0; ^⁑5^*P* < 0.01 vs PEEP5

**Fig. 1:**
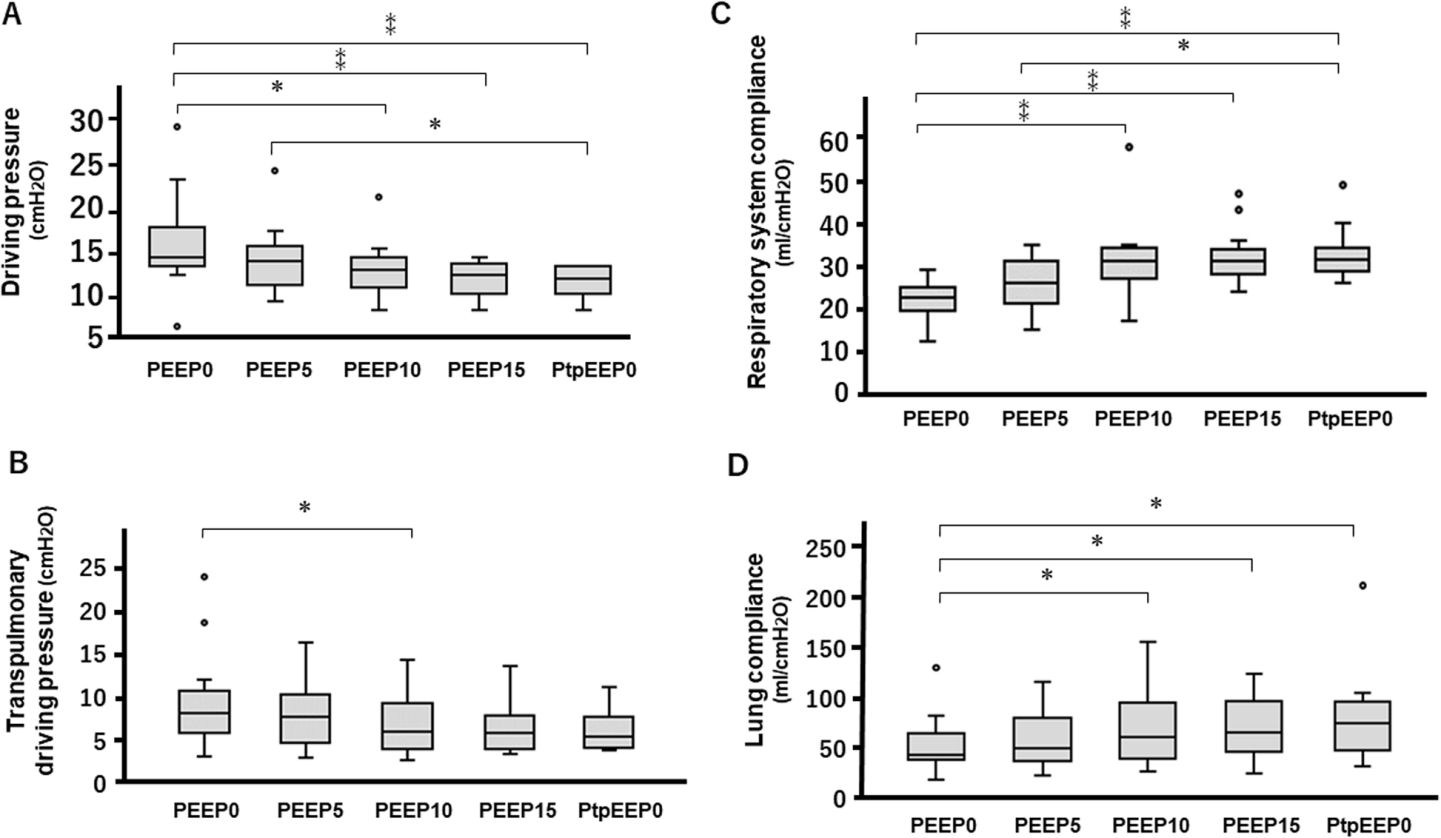
Effects of increasing PEEP on driving pressure (**A**), transpulmonary driving pressure (**B**), respiratory system compliance (**C**), and lung compliance (**D**) during robot assisted laparoscopic prostatectomy. *PtpEEP0* PEEP levels corresponding to the end-expiratory transpulmonary pressure levels of ≥0 cmH_2_O were achieved. *P < 0.05, ⁑*P* < 0.01

The values of DP decreased with the increase in PEEP. The values of DP at PEEP10 (*P* = 0.012), PEEP15 (*P* = 0.001), and PtpEEP0 (*P* < 0.001) were significantly lower than those at PEEP0; those at PtpEEP0 (*P* = 0.036) were also lower than those at PEEP5 (Fig. 1A). PtpDP decreased with the increase in PEEP from PEEP0 to PtpEEP0; however, this change was not significant except for the values between PEEP0 and PEEP10 (Fig. 1B). Respiratory system compliance values were higher at PEEP10, PEEP15, and PtpEEP0 than at PEEP0 (*P* < 0.001); in addition, the corresponding values at PtpEEP0 were higher than those at PEEP5 (*P* = 0.02) (Fig. 1C). The values of lung compliance at PEEP10 (*P* = 0.0118), PEEP15 (*P* = 0.0284), and PtpEEP0 (*P* = 0.0169) were significantly higher than those at PEEP0 (Fig. 1D). Chest wall compliance values were similar at all PEEP levels.

### Arterial blood gas analysis

Blood gas data are shown in Table 3. The values of PaO_2_ at PEEP of ≥10 cmH_2_O were significantly higher than those at PEEP0 (*P* < 0.0024 at PEEP10, *P* < 0.001 at PEEP15, and *P* < 0.001 at PtpEEP0, respectively). The values of PaCO_2_ were higher at PEEP 15 cmH_2_O than at PEEP0 (*P* = 0.0034) and PEEP5 (*P* = 0.0324). The values of pH decreased with the increase in PEEP; those at PtpEEP0 were significantly lower than those at PEEP0 (*P* < 0.001) and PEEP5 (*P* = 0.0324).

**Table 3:** Changes in blood gas data during anesthesia

	After induction	Positive-pressure capnoperitoneum and Trendelenburg position					End of surgery
		PEEP0	PEEP5	PEEP10	PEEP15	PtpEEP0	
pH	7.36 [7.35, 7.38]	7.34 [7.32, 7.36]	7.32 [7.31, 7.35]	7.31 [7.29, 7.33]	7.28 [7.27, 7.33]^⁑0^	7.28 [7.27, 7.33]^⁑0,*5^	7.31 [7.28, 7.34]
PaCO_2_ (mmHg)	44 [42, 46]	50 [45, 52]	52 [47, 55]	50 [49, 51]	55 [49, 59]^⁑0,*5^	52 [48, 61]	54 [52, 57]
PaO_2_ (mmHg)	220 [137, 251]	179 [120, 200]	198 [140, 230]	200 [160, 250]^⁑0^	208 [163, 250]^⁑0^	211 [162, 236]^⁑0^	213 [191, 255]
ETCO_2_ (mmHg)	39 [38, 39]	39 [38, 41]	42 [40, 43]	43 [39, 46]	43 [40, 48]	45 [42, 48] ^*0^	43 [42, 48]

Data are presented as median values [interquartile range]

*PaCO*_*2*_ partial pressure of carbon dioxide, *PEEP* positive end-expiratory pressure

^*0^*P* < 0.05 vs. PEEP0; ^*5^*P* < 0.05 vs. PEEP5; ^⁑0^*P* < 0.01 vs. PEEP0

### Circulatory parameters and cerebral oxygen saturation

Hemodynamic and cerebral oxygenation parameters during anesthesia are presented in Table 4. Heart rate, mean blood pressure, and CI values did not change during anesthesia, except that the values of mean blood pressure at PEEP15 were significantly lower than those at PEEP0 (*P* = 0.0324). The levels of lactate were significantly lower at PEEP15 than at PEEP5 (*P* = 0.032). The values of rSO_2_ at PtpEEP0 were significantly higher than those at PEEP0 (right: *P* = 0.0019, left: *P* = 0.036). There were no differences in the circulatory parameters or rSO_2_ between PEEP5 and PtpEEP0.

**Table 4:** Changes in hemodynamic and cerebral oxygenation parameters during anesthesia

	After induction	Positive-pressure capnoperitoneum and Trendelenburg position					End of surgery
		PEEP0	PEEP5	PEEP10	PEEP15	PtpEEP0	
Heart rate (bpm)	62 [60, 68]	62 [55, 66]	60 [57, 64]	60 [56, 63]	60 [55, 64]	62 [57, 66]	64 [55, 71]
mBP (mmHg)	78 [69, 80]	90 [79, 98]	80 [76, 88]	83 [73, 92]	77 [71, 83] ^*0^	80 [77, 83]	77 [66, 85]
CI (L/min/m^2^)	1.9 [1.7, 2.3]	1.9 [1.8, 2.2]	2 [1.7, 2.1]	1.8 [1.7, 1.9]	1.6 [1.5, 1.9]	1.9 [1.6, 2]	2.3 [2, 2.9]
rSO_2_(rt) (%)	64 [62, 68]	69 [65, 71]	69 [67, 73]	70 [68, 75]	72 [68, 74]	73 [70, 75]^*0^	72 [68, 74]
rSO_2_(lt) (%)	68 [67, 70]	69 [63, 75]	69 [67, 76]	71 [66, 77]	71 [67, 77]	73 [69, 78] ^*0^	74 [68, 77]

*mBP* mean blood pressure, *CI* cardiac index, *rSO*_*2*_ regional cerebral oxygen saturation, *PEEP* positive end-expiratory pressure

^*0^*P* < 0.05 vs. PEEP0

## Discussion

This observational interventional study showed that PesoEEP, suggesting intrathoracic pressure, at end-expiration during capnoperitoneum with Trendelenburg position reached 10 cmH_2_O, and the PEEP levels, where PtpEEP exceeding 0 cmH_2_O, were variable among the patients. Theoretically, PtpEEP should exceed 0 cmH_2_O to open alveolus and minimize the risk of atelectasis. We assumed that PEEP levels to achieve a transpulmonary pressure of 0 cmH_2_O at end-expiration might be enough to prevent atelectasis. The present findings suggest that adding PEEP based on transpulmonary pressure during the RALP procedure may be feasible, as respiratory system compliance and driving pressure at such levels were improved compared to those observed at PEEP5 without disturbing circulatory or cerebral oxygenation parameters.

As shown previously [8–10], our study proved that capnoperitoneum in the Trendelenburg position did not significantly affect cardiac output. In addition, cardiac output was maintained at all PEEP levels. This could be partly accomplished by fluid loading according to the SVV values. On the other hand, positive-pressure capnoperitoneum combined with the Trendelenburg position has been associated with intracranial pressure (ICP) elevation [11]; an increase in thoracic pressure is partially transmitted to central venous pressure (CVP) and may thus increase venous downstream pressure of the brain. The effect of PEEP on ICP yielded conflicting results [12, 13]. Caricato et al. showed that for patients with low respiratory system compliance especially for less compliant lung, cerebral hemodynamics and ICP were not influenced by administration of higher PEEP [12]. On the other hand, Boone et al. reported that a statistically significant relationship between PEEP and ICP or PEEP and CPP was not found in normal lungs or mild to moderate lung injury, but only in severe lung injury [13]. The complex interaction between mechanical ventilation and cerebral hemodynamics appears to be influenced by multiple patient-specific factors. The transmission of PEEP into the thoracic cavity is variable and dependent on the properties of the chest wall and lungs. We did not measure CVP or jugular vein pressure, so it is beyond the scope of the present study. The results of the present study showed that cerebral oxygenation was maintained during incremental PEEP levels. Hypercapnia due to capnoperitoneum in combination with low tidal volume ventilation might contribute to maintain cerebral blood flow and rSO_2_.

Recently, several clinical studies have reported on using an esophageal catheter during anesthetic management of laparoscopic surgery [14–16]. Tharp et al. examined the optimal positive end-expiratory pressure settings needed to achieve positive end-expiratory transpulmonary pressures during robotic laparoscopic surgery, and they showed that body habitus, pneumoperitoneum, and Trendelenburg positioning may each independently impair lung mechanics [14]. Therefore, it seems important that PEEP settings should be individually adjusted based on the variability in surgical conditions and patient’s physique. Piriyapatsom examined the optimal PEEP levels during laparoscopic gynecological surgery, using an esophageal balloon catheter [15]. In this study, the titrated PEEP levels exceeded pleural pressure values. Compared with those at the conventional PEEP level (5 cmH_2_O), respiratory system compliance was higher and driving pressure was lower in the intervention group than in the control group. Those findings were almost identical to ours.

Using an esophageal catheter for measuring esophageal pressure can separate the respiratory mechanics between that of the chest wall and that of the lung, and it provides information about individual lung mechanics, including lung compliance or transpulmonary driving pressure values. Contrary to our expectations, the decrease in transpulmonary driving pressure and the increase in lung compliance by PtpEEP0 were not significant compared to those obtained at PEEP5. We assumed that those higher PEEP levels might not necessarily lead to uniform ventilation. These findings suggest that transpulmonary pressure, estimated by using the esophageal pressure values in the present study, reflects the levels in the dependent lung. Thus, those PEEP values may not be suitable for the entire lung, specifically, for the non-dependent lung, as the distribution of pleural pressure is not homogeneous [17]. Pleural pressure is overestimated in the non-dependent pleural space and underestimated in the dependent regions. Experimental studies have shown the presence of a vertical gradient of pleural pressure from top to bottom [18], which may be related to gravity, the weight of the lungs, and pressure from mediastinal and abdominal contents. In the present study, high PEEP increased PaCO_2_ levels despite increasing minute ventilation while maintaining ETCO_2_ constant, suggesting that the dead space-to-tidal volume ratio might increase. While tidal volume was small and kept constant throughout the surgical procedures, the more compliant lung may be ventilated at a higher PEEP level.

Shono et al. compared PEEP of 15 cmH_2_O with PEEP of 5 cmH_2_O during RALP and demonstrated that PEEP of 15 cmH_2_O might maintain ventilation in the dorsal region and improve lung mechanics using transpulmonary pressure and electrical impedance tomography imaging [16]. These authors concluded that PEEP of 15 cmH_2_O resulted in more homogeneous ventilation and favorable physiologic effects during RALP. This study did not show optimal PEEP levels during RALP; however, the PEEP level of 15 cmH_2_O presented with results comparable to those obtained in the present study.

The present study has several limitations. First, the esophageal pressure can reflect pleural pressure in the regions where the esophageal balloon is located, i.e., mid-lung only if the calibration of the esophageal balloon is performed correctly with minimal non-stress volume. Esophageal pressure can overestimate (too large balloon volume) or underestimate (too small balloon volume) pleural pressure, depending on the volume of the balloon [19]. The inflated volume was automatically adjusted in the present ventilator, and calibration was carried out every 30 min. We ensured that the estimates were based on tidal changes in esophageal pressure. Second, the present study was observational. Randomized controlled studies of the standard PEEP and PEEP at a level above PtpEEP0 are required to validate the present findings. In addition, our findings on the effects of short-term interval PEEP increment may not be suitable for routine clinical practice. Third, esophageal pressure monitoring requires esophageal balloon catheter placement, and it is not practical in the anesthetic management of RALP surgery. The PEEP settings based on transpulmonary pressure measurements are cumbersome and may be unsuitable. Fourth, the present study did not examine the effectiveness of PEEP at a level beyond PtpEEP0. The objective of the present study was to observe the changes in esophageal pressure and PtpEEP during the RALP procedure, depending on the increase in PEEP; this study has achieved its objective.

## Conclusions

Individualized level of PEEP should be determined in patients undergoing the RALP procedure to account for patient and surgical condition heterogeneity. Transpulmonary pressure measurement may help determine optimal PEEP level; however, this approach is not practical and whether thus derived PEEP levels are optimal remains unclear.

## Data Availability

The data that support the findings of this study are available from the corresponding author, KN, upon reasonable request.

## Abbreviations

ASA: American Society of Anesthesiologists
BMI: Body mass index
CI: Cardiac index
CVP: Central venous pressure
FIO_2_: Fraction of inspired oxygen
ICP: Intracranial pressure
PEEP: Positive end-expiratory pressure
PEEP0: PEEP of 0 cmH_2_O
PEEP5: PEEP of 5 cmH_2_O
PEEP10: PEEP of 10 cmH_2_O
PesoEEP: End-expiratory esophageal pressure
PesoEIP: End-inspiratory esophageal pressure
Pplat: Inspiratory plateau pressure
PSi: Patient state index
PtpEEP: End-expiratory transpulmonary pressure
PtpEEP0: PEEP levels corresponding to PtpEEP of 0 cmH_2_O
RALP: Robot-assisted laparoscopic prostatectomy
rSO_2_: Forebrain oxygenation
SVV: Stroke volume variation
VT: Tidal volume
